# The use of predictive models to optimize risk of decisions^☆^

**DOI:** 10.1016/j.ijfoodmicro.2016.10.016

**Published:** 2017-01-02

**Authors:** József Baranyi, Nathália Buss da Silva

**Affiliations:** a Gut Health and Food Safety, Institute of Food Research, Norwich Research Park, Colney Ln, Norwich NR4 7UA, United Kingdom; b Department of Chemical and Food Engineering, Federal University of Santa Catarina, Florianópolis, SC, Brazil

**Keywords:** Predictive microbiology, Bayesian modelling, Decision analysis, Risk assessment

## Abstract

The purpose of this paper is to set up a mathematical framework that risk assessors and regulators could use to quantify the “riskiness” of a particular recommendation (choice/decision). The mathematical theory introduced here can be used for decision support systems. We point out that efficient use of predictive models in decision making for food microbiology needs to consider three major points: (1) the uncertainty and variability of the used information based on which the decision is to be made; (2) the validity of the predictive models aiding the assessor; and (3) the cost generated by the difference between the *a-priory* choice and the *a-posteriori* outcome.

## Highlights

• Predictive models estimate expected responses and their probability distributions derived from a-priori observations.
• Risk of decision is the expected cost of randomly occurring decision errors. This quantification is far from trivial.
• Decision makers should minimize the cost function, however this may produce counter-intuitive results.
• To develop appropriate cost functions to quantify decision errors is as important as to develop predictors.

## 1 Introduction

Predictive food microbiology focuses on the responses of foodborne bacteria to their environment. Sufficiently accurate predictions on bacterial growth and survival can reduce the need for microbiological testing of food, making product formulation and risk assessment much cheaper and more efficient, and ultimately improving microbiological food safety (Ross and McMeekin, 2003). The knowledge gained in the *c.a.* 30 years-long history of the discipline has been implemented in practical decision-supporting software packages, to be used by a range of stakeholders, including industrialists, academicians and regulation officers. Various predictive models are available for different foodborne pathogenic as well as spoilage organisms to help quantitative microbial risk assessment of food (Whiting and Buchanan, 2001, Koutsoumanis et al., 2016).

Practical users face the question to what extent they can rely on the predictions generated by predictive tools, for which one of the most used examples is the ComBase Predictor (ComBase, 2016). Overestimating the growth potentials of pathogenic bacteria can result in food waste and economic loss, while underestimation can have even more serious health- or reputation-related implications (Guillier et al., 2016). Prediction errors can originate from (i) biological and environmental variability; (ii) the uncertainty of the information (observations) on which the predictions are based; and (iii) the inaccuracy of the mathematical models and assumptions used.

Predictive models are predominantly based on simplifying assumptions and observed data. There is no recipe or algorithm to decide whether those simplifying assumptions are valid or allowed; they are accepted if observations validate them (empirical considerations) and they can be embedded in fundamental theories of science (mechanistic reasons). Empirical models are less applicable for extrapolation than mechanistic ones. However, to some degree, all predictions are extrapolations. Mathematical models exist in an ideal Platonian space, from which the applications to future scenarios are inferences. The experimental conditions of the observations, on which the models are based, can rarely be repeated exactly, due to the “*panta rhei*” Heraclitean principle: “*One cannot step into the same water twice*”.

It is relatively easy to test whether a prediction is a mathematical extrapolation; i.e. whether it is outside the range of observations (Baranyi et al., 1999). It is much more difficult to decide whether, for instance, a set of measurements on a proxy organism can be applied to the “real one”. An example for this is applying observations on *Listeria innocua* to infer the kinetics of *Listeria monocytogenes*. Similarly: are certain environmental conditions like food structure, native flora, processing background, etc. negligible? It is also an open question what details of experimental results should be used for a practical predictive tool. Namely, the higher its resolution, i.e. the more explanatory factors and in wider ranges are considered, the less robust the predictive model will be, more pruned to be affected by random errors. Finding a trade-off between resolution and robustness is a central question in predictive modelling (Ratkowsky, 1993).

While acknowledging the importance of such concerns, decision makers may come across even more complex questions when using predictive packages. Should a decision solely rely on predictions, which normally represent the expected value of a response variable in question? A simple method to correct predictions by a “bias factor” was proposed by Ross (1996), refined by Baranyi et al. (1999). It is evident, for example, that conservative (cautious) decisions are useful when the price of the prediction error is high. In fact, as we show below, one faces three major challenges when making decisions based on microbial risk assessment:

i) The accuracy (uncertainty and variability) of observations used for developing the predictive model is not necessarily known or easy to estimate;
ii) Available software packages are primarily based on empirical models and can generate markedly different predictions especially close to extrapolation regions;
iii) It is not straightforward to decide what measure of dissimilarity between prediction and actual response should be used. The cost (either measurable financially, or in damage of reputation, or severity of illnesses, or in decrease of influence or power, etc.) assigned to their individual components are frequently on mixed and asymmetric scales, which makes a combined optimization difficult.

In this paper we explain, backed by examples, why predictive models should be used in combination with a cost-benefit assessment. We point out that a correct strategy does not necessarily focus on the most probable event, but on mitigating the implications of wrong decisions that can randomly and/or sooner or later inevitably occur.

## 2 Theory and examples

It is commonly accepted and frequently cited (WHO/FAO, 2009) that risk assessment consists of four steps: hazard identification, dose-response assessment, exposure assessment and risk characterisation. Microbial risk itself is defined as the probability of an event and the severity of its known or potential adverse health effects. Therefore, mathematically, risk is a two dimensional vector variable assigned to an event. Its components are the probability and the severity of the event. Sometimes the product of the two is called the risk, which can be considered as the “expected severity” of the event. The severity can be quantified by various ways: e.g. number of death/hospitalization; missed working hours; cost of treatments, etc.

The focus of this paper is *not* the above interpreted risk, assigned to an *a-posteriori* event, but the risk of an *a-priory* decision, that we also call choice or bet in what follows. We will suggest and exemplify a formal mathematical definition for the risk of a decision. Its construction aims at the use of predictive microbiology in decision making, when for example a risk assessor needs to provide a recommendation or a regulatory unit or a health worker needs to choose: against what possible future events should be protective measurements introduced.

Assume that a set of information quantified by an **x** random variable (an *n*-dimensional vector) is available on the past behaviour and the present state of a system. A decision maker needs to put forward a guess **b** (also called choice or “bet” in what follows) on a future event in the system, which is quantified by an *m*-dimensional random variable, **y**. To help the decision, an **y** ≈ **g**(**x**) mapping or algorithm (the predictor), based on a mathematical model, is available that allows the estimation of the **y** outcome. Our focus is the error that **b** is not necessarily equal to **y**. The objective is to find a **b**_**opt**_, the “best bet”, which is optimal from a certain point of view.

Obviously, any **b** choice, if based on a reasonable strategy, should depend on (i) the available information expressed by **x** (measurements, observations, with their probability distributions); (ii) the way how the **y** outcome and its probability distribution depends on the past and present of the system (this is approximated by the **g**(**x**) predictor); (iii) the implications if the outcome is different from the guessed one.

Therefore, the typical elements of our task are:

i) Quantify the uncertainty of the information available;
ii) Determine a mathematical model to estimate the outcome **y** as a function of the past behaviour and the present state of the system;
iii) Assign cost to the error generated by the difference between **b**, the a-priori decision (bet) and **y**, the in-fact to be happening *a-posteriori* outcome.

All these variables could also contain time-dependent components, in which case they are stochastic processes (dynamic, **x**(t), **b**(t), **y**(t) variables rather than just static ones). The available information can be a collection of measurements such as data on (possibly time-dependent) bacterial concentrations, or growth/death rates as a function of environmental factors.

The predictor **g**(**x**) is *unbiased* if the expected value of **g**(**x**) is equal to the expected value of **y**. A well-known example for such predictor is when **x** is a set of independent, identically distributed measurements and the **g**(**x**) mapping is the procedure of taking their arithmetical average. A reasonable **b** bet on the result of the next measurement could be this arithmetical average. Note that this number may not be measured, therefore the bet could always be wrong, if for instance the set of possible outcomes consist of discrete values that do not include the calculated average; still the expected difference between the bet and the outcome could be smaller than betting on an outcome that can really occur.

For an example, for the simple *m* = 1 case, consider a “head or tail” trial, with not necessarily equal probabilities for the two possible outcomes, scored by 0 and 1, respectively. What is the best bet for the result of the next toss if the cost of a wrong bet depends on the difference between the bet and the actual outcome? Note that the decision can nominate any real number, not only 0 or 1.

We will see that if the aim is to minimize the expected cost of the error and this cost is proportional to the squared distance between the bet and the outcome, then the “best bet” is the average of the so-far observed experimental results. So though this strategy can never bring a correct prediction, since the decision is a number between 0 and 1, while a single outcome is either 0 or 1; still, in the long run, it leads to minimizing the loss due to wrong decisions.

That **g**(**x**) should be unbiased, i.e. the expected value of **g**(**x**) should be equal to the expected value of **y**, is a rather trivial requirement. Could we impose more restrictions on **g**? For example, what would be the “best bet” if we introduced asymmetric penalties for under- and over-estimations?

Below we define the risk of a decision. Let **b** be a bet on the **y** outcome. Introduce a

$$c\left(b,y\right):R^{m}\times R^{m}\Rightarrow R$$

cost function to quantify the price we would pay for a decision error, where R is the set of real numbers and R^m^ is the set of m-dimensional vectors with components from R. Define the risk of decision **b** as the expected cost caused by the difference between **b** and the outcome **y**, where the expectation (an integral) is calculated as **y** runs through its possible values with *p_y_* probability distribution:

$$\mathrm{Risk}\left(b\right)=E\left(c\left(b,y\right)\right)=\int c\left(b,y\right){\mathrm{dp}}_{y}$$

We claim that this definition for the risk of the decision **b** is suitable for our purposes. The same idea is used for example in pattern recognition (Devroye et al., 1996).

**Fig. 1:**
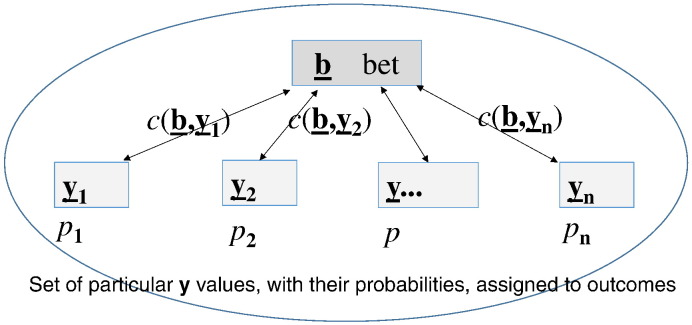
Risk of the decision betting on **b** is determined by the *cost* generated by the differences between **b** and the **y** possible outcomes, and the p(**y**) *probability distribution* assigned to those outcomes.

Fig. 1 demonstrates well the main point this paper addresses; while traditional microbial risk assessment focuses on the *risk of future events*, we concentrate on the *risk of a decision* to be taken *before* those events.

When using an **x** dataset for a-priori information and a **g**(**x**) predictor to estimate **y**, let

$${\mathrm{risk}}_{g}\left(b|x\right)=E\left(c\left(b,g\left(x\right)\right)\right)=\int c\left(b,g\left(x\right)\right)\;dp_{g}$$

be called the *standard estimated* risk, analogously to the standard error in regression. That is: the standard estimated risk of the decision, given a predictor **g**(**x**) for the outcome, is the expected value of the cost generated by the difference between the decision and the predicted outcome. Therefore the estimated risk depends on the available observations (including their probability distributions), the predictive model and the cost of the difference between **b** and the **g**(**x**) prediction. It is trivial that, with sufficiently accurate predictor, the standard estimated risk is expected to be somewhat smaller than the “real” risk, since it does not count with the difference between the prediction and the actual outcome. Its use is that, under natural conditions, the minimum of the standard risk (that can be calculated from available data) should be at the same **b**_**opt**_ bet where also *Risk*(**b**) is optimal.

### 2.1 Two commonly used cost functions applied to a discrete event-space

Our aim is to analyse the nature of the “best bet”, that minimizes the risk of the decision. Here we demonstrate how small changes in the *c* cost function can affect this optimal decision.

1. Suppose that the cost function is proportional to the sum of the squares of the differences between the respective components of the **b** bet and the **y** outcome. This is the basis of the standard least-squares method to fit data. It is usual to call this “the squared distance in L2-norm”, which justifies the notation below

$$c\left(b,y\right)\sim ∥b-y{{∥}^{2}}_{L2}=\sum_{i=1}^{m}{\left(b_{i}-y_{i}\right)}^{2}$$

In this case the risk of the decision **b** is defined as

$$\mathrm{Risk}\left(b\right)=\mathrm{kE}(∥b-y){{∥}^{2}}_{L2})$$

where *k* is a proportionality constant (it does not have any effect on the optimal value **b**_**opt**_).

It is known from regression analysis (Kumar and Singh, 2015) that this risk is minimal if the decision is to bet on the expected value of the outcome

$$b_{\mathrm{opt}\left(L2\right)}=E\left(y\right)$$

Therefore if an unbiased predictive model is available to estimate the outcome, then it provides a minimum-risk decision, assuming that the cost of error is proportional to the sum of squares of the differences between the respective components of the decision and the outcome (Small, 1990). Note that generally many unbiased **g**(**x**) predictors exist and the above statement holds for various **g** predictors if their expected value is equal to the expected outcome.

**Fig. 2:**
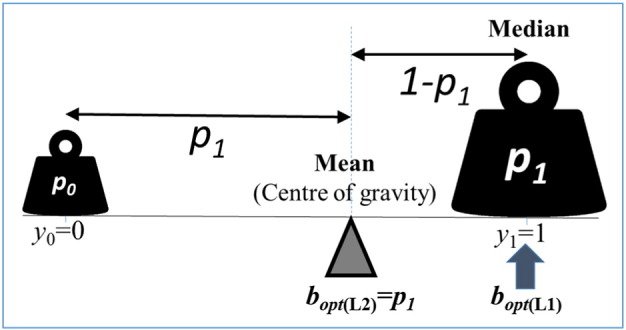
Defining risk by a cost function proportional to the sum of the squares of the difference between bet and outcome (L2-norm) results in the mean (the “centre of gravity”) being the best bet. Replacing the squared-difference with the absolute difference (L1-norm) results in the median of the distribution, which, in our binary case, is the outcome with the highest probability.

A simple demonstration for this, in one dimension (*n* = 1), is shown in Fig. 2, which represents the “head or tail” system with an asymmetric coin. Here the outcome is a single binary random variable **y**, with **y**_0_ = 0 and **y**_1_ = 1 possible values and *p*_0_, *p*_1_ respective probabilities, where *p*_0_ = 1 − *p*_1_, so the mean value is E(**y**) = *p*_1_. What is the best strategy to estimate the outcome of the next toss, given a set of observations in the past?

Imagine that we repeat the decision many times and we need a strategy that is optimal in the long run. What is the best decision to predict the outcome of the next toss? The value of **y** can only be 0 or 1 but the bet can be any real number. Somewhat paradoxically, the best bet is the relative frequency (generated by the **g** predictor from past data) of the “1” results, which is an approximation of *p*_1_. This *p*_1_ can never be an outcome unless *p*_1_ is 0 or 1, which are extreme cases. Normally this bet will never predict the outcome accurately, but the expected cost of its error is the smallest.

The result however that “the best bet is the mean” is true only if the cost is proportional to the squared difference between the bet and the outcome (Fig. 2). If the cost is proportional to the absolute difference between the respective components (and not to the square of those), then we need to find the minimum of

$$\mathrm{Risk}\left(b\right)=E\left({\left\|b-y\right\|}_{L1}\right)$$

In our binary example above, this optimization is equivalent to finding the **b** point on the [0,1] interval where

$$\mathrm{Risk}\left(b\right)=b\cdot \left(1-p_{1}\right)+\left(1-b\right)\cdot p_{1}$$

is minimum. It is an easy exercise to prove that here the risk is minimum if we bet on the event of the higher probability

$$b_{\mathrm{opt}}=0\;\text{if}\;p_{1}<0.5\;\mathrm{and}\;b_{\mathrm{opt}}=1\;\text{if}\;p_{1}>0.5$$

For *p*_0_ = 1/2 = *p*_1_, there is no absolute minimum, the risk is the same for any decision.

This result says that, for binary outcomes (like yes or no), if the cost function is generated by the L1-norm, then the best strategy is to bet on the most probable event. However, if the cost function is generated by the L2-norm then the best bet is a compromise, the mean value. The two cost functions do not seem very different, they both can be conceived and supported by intuition, still they suggest very different strategies for optimal decision.

It can be proven that, for L1-generated cost, the best bet is the median (Kumar and Singh, 2015). One may get the impression that, if the risk is defined in L1-sense, then we should always bet on the median. This conclusion however is true only for one-dimensional events. There is no universal solution for multi-dimension. On the other hand, if the cost is defined in L2-sense then the best bet is always the estimated mean, in higher dimensions, too (Small, 1990).

**Fig. 3:**
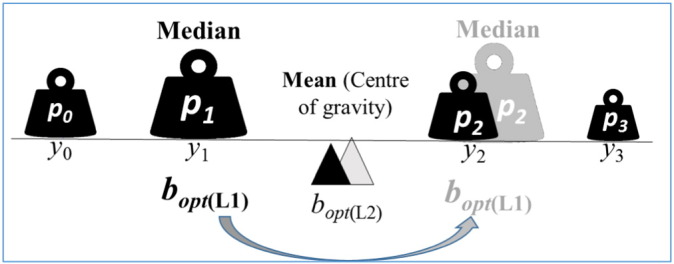
The L1-generated best bet is at the median of the response. It can change abruptly from one possible outcome to the other (unlike the L2-generated best bet) as the probability distribution of the possible outcomes changes continuously.

A consequence is that the median is not a continuous function of the probability weights, which is rather counter-intuitive for a risk function. For example, if the probability of an outcome is gradually increasing, then the median of all (discreet) outcomes can suddenly change from one point to the other. This is demonstrated on Fig. 3. I can be easily shown by induction, for multi-dimension, too, that the L1-minimized risk changes stepwise as the probability distributions change continuously.

**Fig. 4:**
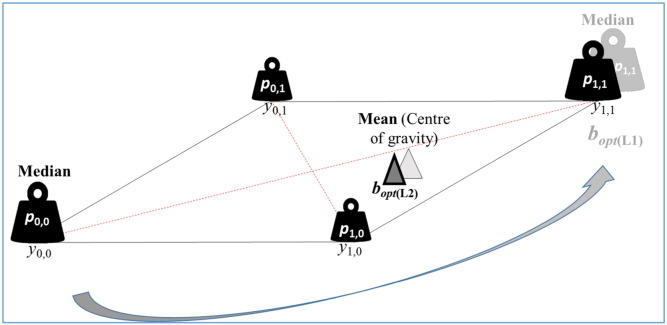
In multi-dimension, the median (L1-generated risk) can be defined unambiguously only under symmetry conditions when the problem is reducible to one dimension (the diagonal in this case).

For an example of the multi-dimension case, let *m* = 2 (i.e. **y** = [**y**_1_**y**_2_]) where **y**_*i*_ are binary random variables with {0,1} possible values. Let *p*_*i*,*j*_ denote the probability of the event **y** = [*i*,*j*] where the sum of the *p*_*i*,*j*_ probabilities (*i* = 0,1; *j* = 0,1) is 1. As follows from above, **b**_**opt**(L2)_ = [*p*_1,0_, *p*_0,1_] (see Fig. 4). How about the other distance-dependent strategies? Intuitively, if *p*_1,0_ = *p*_0,1_ then the problem is symmetric on the (0,0)–(1,1) diagonal therefore the least risky decision also needs to be on it. This way, some results of the one-dimension-case can be applied to this scenario. Like in one-dimension, the L1-optimum still changes stage-wise as the probability weights change continuously. On the other hand, if the probabilities are uniformly distributed, then there IS a unique solution for the optimum (the centre of gravity in the probability space is minimum in this case for both L2- and L1-risk).

### 2.2 Asymmetric cost function on continuous event-space

Some of these examples for risk minimization exhibit stage-wise behaviour as the distribution of the underlying probabilities gradually changes. This was shown for the relatively simple case when the event-space was discrete and the cost of prediction is symmetric; i.e. bets resulting in over- or underestimations had the same distance-measure, therefore the same cost. In food safety and shelf-life studies, the cost function is rarely symmetric; this is why conservative (safe) predictions are preferred (Guillier et al., 2016). This causes further complications in the optimization problem discussed in our paper. We demonstrate this by an example below, now using continuous event-space.

**Fig. 5:**
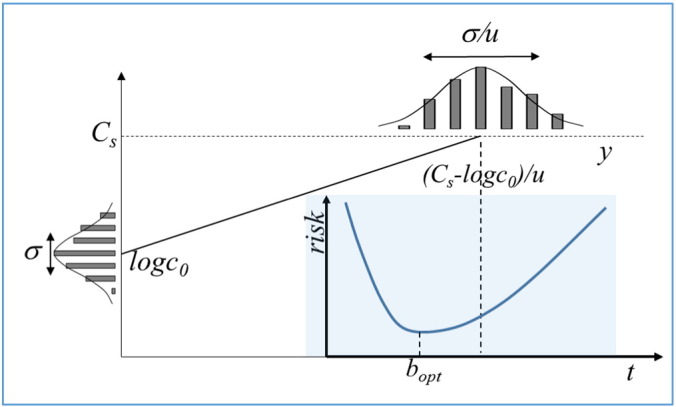
The case of asymmetric cost function. Overestimating the time when a food product is to be withdrawn is less costly (though increases food waste) than underestimating it, which is a health issue.

In a given perishable food product, let the log_10_-concentration of spoilage bacteria, at the time of putting the food packs on the shelf, be normally distributed with log*c*_0_ expected value and *σ* standard deviation. Suppose that the bacteria grow at *u* rate on the log_10_ scale. Then the **y** critical time, when they reach the *C*_*s*_ concentration level (which is a threshold to be unacceptable) is a random variable also following the normal distribution, with (*C*_*s*_ − log*c*_0_) / *u* expected value and *σ*/*u* standard deviation (Fig. 5). The values of these parameters are estimated by means of a set of observations, **x**. What is the optimal time, **b**, to take the packs off the shelf to optimize the risk that either spoiled food could be sold or healthy food could be wasted?

This is the case of asymmetric cost function. Namely, if the food packs are taken off too early (**b** < **y**), that means economic loss and food waste. If they are left on the shelf for too long (**b** > **y**), that can result in illnesses and damage in reputation, with much more serious cost than in the first case. Therefore, the optimal time to take the food off the shelf is definitely sooner than the (*C*_*s*_ − log*c*_0_) / *u* expected time for the bacteria to reach the *C*_*s*_ level. The **b**_**opt**_ optimum time can be estimated by minimizing

$$\mathrm{Risk}\left(b\right)=\int_{0}^{b}c_{1}\left(b,y\right)\cdot \varphi \left(y\right)\mathrm{dy}+\int_{b}^{\infty }c_{2}\left(b,y\right)\cdot \varphi \left(y\right)\mathrm{dy}$$

where *φ*(**y**) is the probability distribution function of the normal distribution with (*C*_*s*_ − log*c*_0_) / *u* expected value and *σ*/*q* standard deviation; and *c*_*i*_(**b**,**y**) (*i* = 1,2) are the cost functions for the cases **y** < **b** and **b** < **y**, respectively, corresponding to the two parts of the above integral. For example, if the cost of overestimating the critical time (**y** < **b**: food safety issue), is proportional to the difference between the actual bacterial concentration and its allowed 10^*Cs*^ level, then the cost can be estimated by

$$c_{1}\left(b,y\right)=d_{1}\left(10^{\log c0+ub}-10^{\mathrm{Cs}}\right)\;\left(y<b\right)$$

where *d*_1_ is a proportionality constant. On the other hand, for the case when the **b** bet underestimates the critical time (**b** < **y**), it is reasonable to assume that the waiting time for a pack to be bought by a customer follows the exponential distribution, with the parameter *v* on the log_10_ scale, in which case the expected proportion of packs taken off before time is (10^−* v***b**^ − 10^−* v***y**^), with the cost

$$c_{2}\left(b,y\right)=d_{2}\left(10^{-vb}-10^{-vy}\right)\;\left(b<y\right)$$

again with some *d*_2_ proportionality constant. The optimal decision is where the derivative of *Risk*(**b**) becomes zero, as demonstrated in Fig. 5.

## 3 Discussion

We demonstrated that (i) the uncertainty and variability of available information and (ii) the (possibly subjective) choice of the cost function generated by the difference between the bet and the actual outcome are major factors when using predictive models to optimize risk of decisions. While model developers and decision makers are mostly aware of the first factor, the nature and significance of the cost structure is less analysed. Users may expect that the risk (expected cost) associated to a decision is a continuous function of the probability distribution of the observations and we showed that with relatively straightforward cost functions (see the L1-minimized risk) this does not hold. On the other hand, in case of multi-dimensional and/or asymmetric cost functions, their non-uniform scaling may cause difficulties. A simple example shown here was the optimal time for withdrawal of food products from shelves: the cost of underestimation of this time is measurable in economic loss and food waste, while overestimation may result in health-problems for the consumer. Unifying such scales are especially important when one objective function (e.g. food safety) works against another (e.g. food waste; see Guillier et al., 2016). Another example for a two-dimensional case worth mentioning is when food quality is quantified by the concentration of its spoilage organisms and by a score characterising its organoleptic properties. This leads to the scenario when heat treatment improves the microbiological aspects while making the other worse.

As emphasized above here it is not a future event to which risk is assigned but to a current decision. This idea is more related to decision analysis and game theory rather than traditional microbial risk assessment. In game theory, for an agent, the probability distribution of the future move of the opponent helps to find an optimal move. Our scenario is similar: it is the probability distribution of future events what we use to assign an expected cost (quantified consequence or implication) to a decision, by which we define its risk. The analogy can go on, considering that the opponent can also calculate the strategy of this agent, therefore may change his, generating a dynamic feed-back loop. This can also happen for example between supermarkets and consumers. In our last example above, the price of the food product could be decreased in order to encourage its sale, but the consumer may choose to wait for this, which could accelerate the price decrease, again causing a feed-back loop.

Our first example, the binary scenario, resembles the “type I” and “type II” errors in statistical hypothesis testing; the first being the incorrect rejection of a true null hypothesis (a “false positive”, i.e., accepting a false hypothesis as correct), the second being the failure to reject a false null hypothesis (a “false negative”, i.e., rejecting a true hypothesis as incorrect). The decision on acceptance or rejection is commonly made on the basis of a “significance level”, which is a threshold probability that could be rather subjective. A possible objective determination of this level is to decide it on the cost of the two types of error. For example, such a critical decision is made in forensic sciences, typically with the justification that “if innocent, then an event happened which has an extremely low probability”. In western democracies this level is set very low, because unjust sentencing is considered much more serious error than setting guilty ones free.

These analogies and examples intend to highlight, from different angles, the importance of cost-function analysis hand-in-hand with predictive models. Users should be encouraged to think of such analyses rather than accepting predictions as they are. An approach to objective decision making should be to minimize the cost of inevitable errors, while being aware that the result can be highly sensitive to the structure of the cost-functions assigned to those errors.
